# Research progress on the application of Kawakita Jiro Method in nursing field

**DOI:** 10.3389/fmed.2026.1917608

**Published:** 2026-08-17

**Authors:** Xinyi Hao, Jin Guo, Xiaolan Dong

**Affiliations:** 1 Department of Nursing, Peking Union Medical College Hospital, Chinese Academy of Medical Sciences, Beijing, China; 2 Department of Dermatology, Peking Union Medical College Hospital, Chinese Academy of Medical Sciences & Peking Union Medical College, Beijing, China

**Keywords:** affinity diagram, Kawakita Jiro method, KJ method, nursing care, qualitative integration

## Abstract

In recent years, the application of Kawakita Jiro Method (KJ method, also known as Affinity Graph Method) in the field of nursing has received increasing attention. As a creative qualitative problem solving method, it provides systematic information analysis and problem solving ideas for the development of nursing education, nursing management, clinical nursing and nursing research. This review provides a structured overview of the core operation process and application characteristics of KJ method, and comprehensively summarizes its application status in nursing education framework construction, teaching reform exploration, nurse–patient communication optimization in nursing management, core competence mining of nurses, patient experience exploration in clinical nursing, improvement of nurse literacy, theoretical framework construction and research tool development in nursing research. The application fit, specific application strategies and practical application effects of KJ method and each nursing scenario were deeply analyzed, and the advantages and existing problems of KJ method in the nursing field were clarified. The future development direction of KJ method was proposed from four dimensions: interdisciplinary integration, application scenario expansion, methodology innovation and nurse core competence training. This paper aims to provide a valuable and targeted reference for the standardized application, innovative practice and follow-up research of KJ method in the nursing field.

## 1 Introduction

As an important part of the health care system, the nursing field is always committed to improving the quality of patient care, optimizing the nursing process and cultivating high-quality nursing talents. With the rapid development of medical technology and the increasingly diverse needs of patients, the challenges faced by nursing work are more and more complex. Nurses are not only required to have solid professional knowledge and skills, but also to quickly analyze problems, develop solutions, and collaborate efficiently with multidisciplinary teams in complex clinical Settings. In this context, how to systematically organize and analyze information and improve problem solving ability has become a key issue to be solved in the nursing field.

Kawakita Jiro Method (KJ Method) (1), also known as Affinity Graph Method, is a creative problem-solving method proposed by Japanese cultural anthropologist Kawakita Jiro in the 1960s. This method is an important tool for data processing and problem analysis of qualitative research. The core application process is as follows: first, information is collected through interviews, brainstorming, literature combing, etc., and scattered views, data, and experience descriptions obtained are converted into simple text records and made into independent cards. Then, the cards were sorted layer by layer, classified and merged to complete the card grouping. The core connotation of each group of cards was refined and the label was named. Finally, according to the logical relationship between groups, the structure diagram is drawn, and the scattered qualitative information is transformed into a structured knowledge system, so as to help the team or individual accurately understand the problem, discover the internal rules and formulate targeted solutions (2, 3). This method was first used in the collation and analysis of field survey data, and then gradually expanded to many fields such as management, education, engineering design and so on. In recent years, its application scenarios in the field of nursing have been enriched, and its application needs have also changed with the development of nursing disciplines. Methodologically, the KJ approach occupies a unique position within the qualitative research paradigm. Unlike thematic analysis, where themes are extracted through researcher-led coding, the KJ approach emphasizes a bottom-up participatory process in which raw data are organized through physical card sorting and iterative grouping combined with direct stakeholder input (4, 5). Unlike grounded theory, which aims to generate new theories from data, the KJ approach is primarily a meaning-building and consensus building tool that organizes existing information to reveal implied structures. Unlike content analysis, which quantifies category frequencies, the KJ approach is inductive and relational focused, emphasizing logical connections between emerging themes. Thus, the KJ approach is understood as a structured analysis strategy for integrating different qualitative inputs and building conceptual frameworks.

However, the KJ method also has some limitations. For example, its reliance on subjective judgment in the process of card grouping and labeling may introduce bias and affect reproducibility. Secondly, in interdisciplinary or hierarchical team Settings, the degree of cooperation and communication among team members may affect brainstorming and consensus building, thereby potentially reducing effectiveness. It is urgent to systematically review its application process, scene fit, application effect and development direction in the field of nursing. This review aims to systematically sort out the application progress of KJ method in the nursing field, so as to provide a reference for the follow-up research and practical application of this method in the nursing field.

## 2 The research status of KJ method in nursing education

KJ method has become an effective tool for the reform and development of nursing education with its advantages of systematic qualitative information collation and analysis. It can accurately excavate the needs and experience of teaching subjects, comb the logic system of teaching content, collect teaching related qualitative data combined with interview method, extract core themes through card grouping, label naming and other steps, and provide a basis for teaching design and reform. It has shown good application effects in the construction of teaching system and teaching model optimization of fundamental nursing.

### 2.1 Restructure the basic teaching content to help the construction of nursing framework and the design of module system

Within the context of constructing nursing education frameworks, Yitong Zhou and Geng et al. (6) used KJ method to construct the overall framework of basic nursing operation technology, identifying seven content areas including infection control technology, patient data collection and recording, and assisted cleaning and appropriate activity technology. Here, the KJ method’ score advantage was its inductive, bottom-up approach: rather than imposing predetermined categories derived from existing curricula or textbooks, the research team derived themes directly from clinical practice data through card-based grouping, ensuring that the resulting framework genuinely reflected the realities of nursing workflows.

Geng (7) further applied KJ method to systematically analyze practical teaching content, constructing a basic nursing practice teaching system with three first-level modules: “basic principles and methods,” “nursing technology to meet basic needs,” and “nursing technology to meet special needs.” In this study, KJ method’ s visual and collaborative nature proved particularly valuable: the research team—comprising educators, clinicians, and curriculum designers with diverse expertise—could collectively sort, discuss, and rearrange cards on a physical workspace, building consensus on module boundaries and hierarchical relationships in ways that individual coding methods like thematic analysis (8) cannot easily replicate. The resulting framework reflected the autonomy, diversity, hierarchy, priority, and comprehensiveness of basic nursing technology operations, providing a basis for clarifying the overall positioning of technical operations and re-integrating curriculum content.

These two studies illustrate the situations where KJ method is particularly preferable in nursing education research: when the research aims to capture and integrate multiple stakeholder perspectives (educators, students); when the conceptual landscape is exploratory and categories cannot be predetermined; and when team-based consensus-building is essential for translating diverse inputs into a coherent educational framework. However, it is also important to acknowledge what these studies do not fully address, the method’ s effectiveness is heavily dependent on the composition and dynamics of the grouping team—different team compositions may yield different thematic structures, and the method does not provide clear guidance on how to resolve persistent disagreements during consensus-building.

### 2.2 Deeply understand the learning experience and needs, and explore the new mode of teaching reform

Understanding the real experience of learners is the basis of nursing education reform. However, learning experiences are often those in which early learners may possess valuable insights but have difficulty expressing them in a structured way. The KJ method addresses these gaps through three unique features. First, its bottom-up logic allows themes to emerge from the learner’ s narrative, rather than being constrained by preconceptions in exploring new instructional models to which existing theories may not apply. Second, its card-based operation provides a structured and flexible pathway that increases the transparency of analysis: each topic can be traced back to specific cards and raw data, making the process easier to audit than purely narrative-based approaches (2). Third, its visual and collaborative nature helped to build consensus among multiple researchers -the arrangement of physical cards made disagreements visible and resolvable, reducing personal bias.

In the course reform, Yin et al. (9) introduced KJ method into the course of Nursing Interpersonal Communication. This approach serves a dual function -as both a research tool (analyzing interview data) and a teaching tool (helping students organize their thinking) -which is a unique advantage that other qualitative methods do not have. During the epidemic of COVID-19, Yao Li et al. (10) led 15 nursing students to investigate online PBL-CBL teaching. The KJ analysis identified 4 themes, revealing a subtle finding – “enhanced interaction, but demanding high autonomy”-that emerged specifically when cards were grouped, which made thematic tensions visible in ways that might not be seen with traditional summaries. Sun et al. (11) applied the KJ method to evaluate the hybrid flexible curriculum and revealed the interconnections among the three identified themes through visual diagrams. In specialist training, Zhang et al. (12) and Wang et al. (13) used KJ methods to integrate the perspectives of multiple trainees and identify common challenges and individual differences.

However, important limitations remain. Sample sizes ranged from 8 to 15 participants, which is appropriate for in-depth studies but limits generalizability and representativeness. Furthermore, these studies capture self-reported perceptions rather than objective learning outcomes. Claims regarding “effectiveness improvement” should be viewed as perceived benefits, and future research should integrate KJ-derived insights with objective competency assessments to verify whether educational improvements translate into measurable improvements. Despite these limitations, the KJ method has shown clear value as an exploratory, learner-centered analytical tool in nursing education research, as long as its findings are viewed as preliminary and subject to further validation.

## 3 The research status of KJ method in nursing management

Nursing management involves the coordination of nurse–patient relationship, nurses’ career development, nursing model optimization and other complex dimensions, and its work needs to be based on systematic and accurate qualitative information analysis. The fit between KJ method and nursing management scenario is reflected in the ability to conduct structured analysis of scattered information such as nurse–patient communication, nurses’ career development, and nursing unit operation, accurately locate management problems and excavates core needs. The core application strategy was to collect management-related qualitative data through multi-subject interviews, on-site survey and other methods, and to use KJ method to complete the classification of data, theme refinement and logic sorting, so as to provide a basis for management decision-making. It had achieved significant application effects in the maintenance of nurse–patient relationship, the mining of nurses’ core competence, and the optimization of nursing mode.

### 3.1 Explore the difficulties of nurse–patient communication and maintain a harmonious nurse–patient relationship

Nurse–patient communication is critical in nursing work, with influencing factors spanning providers, patients, and environmental contexts. The KJ method is particularly suited to this multidimensional challenge. In understanding communication difficulties, KJ method has been applied primarily as a diagnostic or problem-identification tool, rather than a direct intervention for improving communication. Its contribution lies in systematically organizing fragmented data to reveal patterns that inform subsequent improvement strategies.

Domestic scholars (14) interviewed 10 patients and 10 nurses across diverse specialties, collecting both parties’ experiences and needs. KJ analysis identified key influencing factors: providers’ humanistic care concepts, professional level, and communication ability; and patients’ cultural background, illness severity, and emotional state. The inclusion of both perspectives within a single KJ analysis revealed areas of alignment (both prioritized communication skills) but also critical divergences: nurses emphasized professional constraints, while patients highlighted unmet emotional needs. However, the study did not report consensus procedures or team composition, and the small, single-site sample limits transferability.

Korean scholars (15) investigated pediatric emergency communication challenges through interviews with 11 parents and 11 emergency staff (including security personnel). KJ analysis extracted three core problems: “information asymmetry,” “inappropriate communication timing,” and “non-medical needs not being met timely.” The multi-perspective design—including non-clinical staff—identified barriers extending beyond clinical interactions to administrative and environmental factors, insights likely missed had only clinicians and parents been included. Based on diagnostic findings, an AI-assisted communication agent was proposed, though its effectiveness remains untested.

Methodologically, both studies demonstrate KJ method’s utility in synthesizing complex multi-source data into actionable frameworks. A distinctive feature of KJ method in communication research is its capacity to integrate multiple stakeholder perspectives within a single analytical framework. Unlike approaches that analyze provider and patient data separately—where each group’s transcripts are coded independently—KJ method allows data from different groups to be visually co-located and compared during the grouping stage. This enables researchers to identify not only what each group perceives as problematic, but also where their perceptions converge or diverge—revealing communication breakdowns as relational phenomena rather than problems attributable to one party. This relational insight—the identification of perceptual gaps between groups—is a unique contribution of KJ method difficult to achieve through separate analyses.

In addition, the KJ method’s role should be clearly distinguished: in these studies, it served as a diagnostic tool—systematically revealing factors and problems affecting communication, but it did not improve communication by itself. Claims regarding “improved communication effects” reflect recommendations for future interventions rather than demonstrated outcomes directly attributable to KJ method. Translation of KJ-derived insights into actual improvement would require additional intervention studies with pre-post measurements. Therefore, KJ method’s value in this context should be understood as diagnostic and formative, generating evidence-informed recommendations that require subsequent validation.

### 3.2 Excavating nurses’ career development needs and clarifying the core competencies required by nurses

The professional development needs and core competence framework of specialized nurses is an important research content of nursing management (16). The relevant information mainly comes from the clinical practice experience and career development demands of nurses, which has strong qualitative characteristics. KJ method can accurately excavates the internal needs of nurses’ career development and combs the constituent system and logical relationship of core competence. Within this context, the KJ method serves three interconnected functions: it structures raw interview data into thematic categories through card-based grouping; synthesizes perspectives across participants, making divergences and convergences visible; and visualizes interrelationships among competencies in diagrammatic form for managerial use. Compared with the Delphi method—which builds consensus through iterative expert surveys with numerical aggregation—KJ method preserves richer qualitative detail and reveals relational patterns, but lacks standardized consensus metrics and validity testing procedures.

In a study (17) of stratified nurse training needs, researchers conducted semi-structured interviews with 12 head nurses and 12 staff nurses, using KJ method to synthesize their perceptions, suggestions, and training requirements. The analysis yielded four thematic categories—management factors, training curriculum implementation, stratified training needs, and assessment formats—comprising 10 sub-themes and 46 tertiary themes, providing a granular evidence base for institutional training reform.

Cheng et al. (18) and other scholars selected 14 clinical specialist nurses from different fields in Class iii Grade A hospitals to conduct in-depth interviews on their own career development needs. KJ method was used to analyze and summarize the collected data, and it was clear that the core career development needs of specialist nurses were “improving professional identity,” “improving career management system” and “enriching training forms and contents.” Based on this, it is proposed to enhance the professional identity of specialist nurses, meet their professional management needs, carry out diversified training and other measures, which provides feasible management decision-making reference for promoting the development of specialist nurses and improving the level of specialist nursing. Japanese scholars (19)conducted semi-structured interviews with 8 nurses in the department of obstetrics and gynecology, collected relevant information such as the competencies and working skills required by nurses in clinical practice, and used KJ method to summarize and analyze the data and logically sort out, and accurately refined 7 core competencies required by occupational health nurses: Self-growth ability, nursing essential continuation ability, strategic planning and responsibility performance ability, coordination ability, client growth support ability, team empowerment ability and creative ability, and clearly showed the logical relationship among the seven. It provides a scientific and systematic basis for nursing managers to formulate targeted nurse training plans, carry out personnel ability assessment and plan nurses’ career development. Here, KJ method served as a competency-structuring tool that not only identified discrete competencies but also revealed their interrelationships (e.g., how coordination ability supports team empowerment, which in turn enables client growth support). This relational mapping is a distinctive contribution of KJ method that simpler competency checklists cannot provide.

The studies demonstrate KJ method’ s utility in synthesizing qualitative data into competency frameworks. However, the small sample sizes are a significant limitation. While such samples are appropriate for exploratory, in-depth qualitative inquiry—where the goal is to generate rich descriptions and uncover underlying patterns rather than to achieve statistical representativeness—they necessarily limit the transferability of findings to broader populations. Therefore, KJ-derived frameworks should be viewed as hypothesis-generating models requiring subsequent validation through larger-scale surveys or multi-site studies.

The studies demonstrate KJ method’s utility in synthesizing qualitative data into competency frameworks. However, small sample sizes limit transferability; findings should be viewed as hypothesis-generating models requiring validation through larger-scale surveys or multi-site studies. Cultural and organizational contexts also shape KJ outputs: the Japanese study (19) identified “grateful communication” and “altruism and reciprocity” as foundational competencies—themes reflecting collectivist workplace culture—while the Chinese study (18) prioritized “improving career management systems,” reflecting institutional transition challenges. These frameworks thus require local validation before implementation.

Compared with quantitative surveys or the Delphi method, KJ method excels at capturing emergent, unanticipated themes but offers reduced generalizability and weaker procedural transparency. Despite these limitations, it provides unique value in exploratory competency development when stakeholder perspectives are diverse and relational understanding is essential. When used as a diagnostic and structuring tool with appropriate methodological reporting and subsequent validation, KJ method can contribute meaningfully to evidence-based nursing workforce development.

### 3.3 Improve the nursing unit delivery model and focus on the workplace social capital attributes of nurses

The research on nursing unit delivery model optimization and nurses’ workplace social capital needs to integrate nursing management, clinical practice, nurse experience and other aspects of information. KJ method collects data through multi-subject brainstorming, nurse interviews and other ways, and complete the classification and analysis of KJ method combined with industry norms to ensure that the research results are consistent with nursing clinical practice. American scholar.

Polancich et al. (20) formed a team comprising university faculty, hospital administrators, head nurses, and frontline nurses, using brainstorming and affinity mapping to evaluate the care delivery model of a nursing unit. The team reviewed project tasks according to Nursing Committee regulations, determined the distinct functions of registered nurses and nurse practitioners, and allocated tasks to appropriate providers, generating workforce recommendations expected to reduce workload and improve efficiency. In this study, KJ method functioned as a diagnostic and planning tool—organizing stakeholder perspectives and generating evidence-informed recommendations for redesign. However, actual improvement outcomes remain anticipated rather than confirmed, as post-implementation evaluation data were not reported. Norikoshi et al. (21) interviewed 32 nurses across seven hospitals, collecting data on workplace interactions, teamwork perceptions, and organizational support. KJ analysis synthesized six attributes of workplace social capital: affirmation, grateful communication, information sharing, trust, access to power, and altruism-reciprocity. Here, KJ method served as a conceptual clarification tool—transforming fragmented experiential data into a structured framework for managers to foster positive workplace climates. However, the suggested link from these attributes to high-quality patient services remains theoretical and requires empirical validation.

The reviewed studies demonstrate that KJ method can identify problems, reveal root causes, and generate recommendations—but not that it directly produces measurable quality improvements. Claims regarding “improved efficiency” or “enhanced patient care” should be interpreted as anticipated benefits rather than validated outcomes. Future research should track implementation fidelity and measure actual outcomes to verify whether KJ-derived recommendations translate into tangible quality improvements. Thus, KJ method is best understood as a diagnostic and planning tool rather than a direct quality improvement intervention.

As a structured tool for organizing qualitative information, the KJ method helps nursing managers clarify ambiguous issues by categorizing disparate opinions and observations into an affinity diagram. This process facilitates collective organizational thinking, as it enables diverse perspectives to be integrated through team-based grouping and labeling (22, 23). Consequently, the method supports systematic problem-solving in nursing management by providing a clear structural framework for analyzing complex situations, identifying root causes, and developing targeted improvement strategies (24). It is therefore a valuable tool for stimulating creative thinking and fostering consensus-driven decision-making in clinical settings (25).

## 4 The research status of KJ method in clinical nursing

Clinical nursing work faces the needs of patients directly, involving patients’ disease experience, self-management, nurses’ clinical ability improvement and other aspects. The relevant information is mostly the subjective experience of patients and nurses, and the qualitative problems in clinical practice, with scattered and personalized characteristics. KJ method can accurately excavate patients’ disease experience and nursing needs, locate the shortcomings of nurses’ clinical ability, collect qualitative data by semi-structured interviews and clinical research, and complete the structured analysis and theme refinement of data through the standardized process of KJ method, so as to provide an accurate basis for the formulation of clinical nursing plans and the improvement of nurses’ ability. It has shown good application effects in patients’ health management and nurses’ comprehensive literacy improvement.

### 4.1 Explore patients’ disease experience and coping experience to promote patients’ health management

Exploring patients’ disease experiences and coping strategies is a core task in clinical nursing research. However, patients’ subjective experiences are often fragmented and deeply embedded in individual narratives, posing challenges for conventional qualitative analysis. The KJ method is particularly well-suited to this context for three reasons: its card-based operation distills lengthy narratives into manageable meaning units; its bottom-up grouping allows themes to emerge inductively, capturing unanticipated aspects of illness experiences; and its visual diagramming transforms scattered accounts into coherent pathways that reveal temporal and causal relationships among patients’ perceptions, coping behaviors, and care needs.

In exploring patients’ disease response processes, Shanwen Sun et al. (26) applied the KJ method to investigate how 12 patients with respiratory tract infections recognize symptoms and formulate coping strategies. Five themes were identified and organized into visual pathways, with the KJ method functioning as a process-mapping tool that explicitly revealed the temporal sequence of patient decision-making and identified critical intervention points. Kyoko Nagata et al. (27) explored the subjective experiences of nine community-dwelling schizophrenia patients, extracting ten themes including initial confusion and helplessness. Here, the KJ method served as a theme-structuring tool that prioritized hierarchical relationships among experiential components, revealing that “initial confusion” preceded and potentially triggered subsequent feelings of helplessness. Ee et al. (28) investigated experiences of 21 elderly Dutch breast cancer patients, using open coding combined with affinity mapping. This hybrid approach demonstrated how KJ synthesis revealed interconnections among themes—showing, for instance, how inadequate information exchange directly impacted support experiences.

In identifying care needs and informing intervention design, Huimin Pang (29) interviewed 12 physicians, nurses, and dietitians involved in Parkinson’s disease care, using KJ method to extract five themes that systematically summarized nutritional intervention status. The KJ method functioned as a multi-stakeholder synthesis tool, integrating diverse professional perspectives into a unified problem framework. Xinyi Hao et al. (1) explored self-management preferences among ten recurrent gout patients, identifying seven themes through KJ analysis. Here, the method served as a preference-elucidation tool that organized patients’ self-management challenges into a structured framework directly informing nursing strategies. Guojing Ge (30) conducted a comparative study of hospice care in China and Japan, using KJ method as a cross-cultural synthesis tool that organized diverse qualitative inputs from interviews, ward observations, and conferences into comparable thematic structures, revealing Japan’s established model and China’ s development trajectory.

Across these applications, the KJ method consistently plays three interconnected roles: process-mapping (visualizing temporal sequences of illness responses), theme-structuring (organizing fragmented data into hierarchical frameworks), and needs-identifying (translating perspectives into actionable clinical priorities). Notably, application modalities vary: some studies used KJ as the sole analytical approach, others combined it with pre-existing theoretical models, and still others adopted hybrid approaches with coding followed by affinity mapping—demonstrating the method’s flexibility, provided core principles are maintained. However, the lack of standardized reporting guidelines for such adaptations complicates cross-study comparison and quality assessment, warranting future methodological research.

### 4.2 Explore the shortcomings of clinical nurses’ ability development and promote the comprehensive quality of nurses

The professional ability of clinical nurses is the core factor affecting the quality of nursing, and their shortcomings and improvement needs are mostly hidden in the qualitative experience of clinical practice. KJ method can accurately comb the problems and needs of nurses in clinical practice, locate the core shortcomings of ability development, collect qualitative data through nurses’ clinical practice interviews, and use KJ method to refine the core themes. To provide a basis for the formulation of nurses’ ability improvement programs.

Patient-Reported Outcomes (PRO) is often used in neurosurgery nursing to address unmet needs after discharge. Han et al. (31), explored the perspectives and experiences of neurosurgery nurses to guide the nurse-led PROs system. The researchers conducted offline semi-structured interviews with 15 neurosurgery nurses from two institutions to explore the nurses’ cognition, barriers and practical application of PRO. The analysis of KJ method was used to identify theme patterns and 6 themes were identified: Current challenges, cognitive level, patient’s medical experience, influencing factors, system support, and “shared decision resolution” model. The core shortcomings of neurosurgery nurses in the implementation of PRO were accurately identified as “insufficient cognition, lack of system support, and insufficient multidisciplinary collaboration.” Accordingly, it was proposed that senior management give priority to nurse training, improve digital infrastructure, and strengthen multidisciplinary collaboration, which provided a feasible path for making up for the lack of PRO implementation and improving the quality of neurosurgery nursing. Wang et al. (32) and other scholars designed a qualitative study to evaluate the proficiency of neurology nurses in nursing patients with indwelling nasointestinal tubes, and conducted semi-structured and in-depth interviews with 11 neurology nurses. KJ method was used to refine themes from the results of semi-structured interviews, which were as follows: Preparation and assessment before intubation, increasing the success rate of intubation, prevention of complications, management of complications and emergencies, acquisition of relevant knowledge and experience, and then organizing the topics into coherent visual and logical paths. In clinical practice, nurses’ bedside blind nasointestinal tube placement technology has not yet formed a unified standard. The results of this study show that there are differences in the knowledge and experience of neurology nurses in nasointestinal tube care. In the future, targeted improvement measures can be formulated to improve the quality of patient care.

In addition to clinical nursing skills, carrying out scientific research activities is also one of the comprehensive qualities required by nursing staff. Hongyan Wu et al. (33) selected 10 nurses and conducted in-depth interviews on the influencing factors of scientific research activities. In the process of data analysis, KJ method was used to summarize the theme. The results showed that the influencing factors of nursing research by nursing staff came from three aspects: personal factors such as lack of knowledge, lack of motivation, scientific research collaboration factors and management factors. It can be seen that the influencing factors of nursing research activities are various, and the support for nursing research activities of nurses should be paid attention to subjectively and create conditions objectively.

### 4.3 Research status of KJ method in nursing research

As an important method of qualitative research, KJ method itself is one of the tools of nursing research. Its high fit with the scene of nursing research is reflected in the ability to conduct structural analysis of complex research data, excavate the logical relationship between the data, help the construction of theoretical framework and the development of research tools, and collect research data combined with literature analysis, interview survey, brainstorming and other methods. Through the standardized process of KJ method, the data classification, theme refinement and logic sorting can be completed. At the same time, it can be combined with other research methods to improve the scientific and comprehensive nature of the research. It plays an important role in the theory construction and tool development of nursing research, and the application effect is significant.

### 4.4 Hierarchical classification presents logical relationships and helps to build a theoretical framework

KJ method is to collect language data of different types and majority of people to form more multi-direction and multi-angle different views, understandings and ideas on the same thing, so as to tap the potential of people’s wisdom more deeply. The effective combination of KJ method and “literature reading method,” “brainstorming method,” “system analysis method” and “association graph method” encourages all kinds of people to carry out innovative and bold ideas on things, so as to break the status quo and create new ideas or new models. It can reflect the causality of complex and fuzzy problems more intuitively, thus having strong operability (2).

In various studies, the application of the KJ method follows a consistent logic: collect different qualitative inputs, then organize them through affinity mapping, and finally transform them into conceptual models. However, a rigorous examination revealed significant heterogeneity in the quality of the operations. Researchers at the University of Washington (34) used iterative affinity mapping to categorize 90 usability issues in evidence-based psychosocial interventions into 12 categories and proposed 12 corresponding heuristics. In this study, the KJ method served as a bottom-up, team-based meaning construction tool: researchers physically grouped the cards based on the perceived natural relationships, allowing for the emergence of categories rather than being imposed by a predefined coding scheme. This method is fundamentally different from thematic analysis, where researchers independently code transcripts and iteratively refine themes through systematic reading and re-reading. The collaboration and visibility of the KJ method enable the research team to establish consensus on category boundaries through direct discussion - a process that is difficult to replicate in a single coding. However, this study did not report the agreement among evaluators or the team composition, so it is unclear to what extent the 12 categories structure reflects the real data patterns and team dynamics. Rutkowski et al. (35) pointed out the limitations of the existing “task-oriented” caregiver information behavior model. Through interviews with 30 dementia caregivers, they used team affinity mapping to decompose the information-seeking component into input, process, output, and feedback, forming a comprehensive “process-level” model. This study demonstrated strong methodological transparency by clearly defining the team composition and consensus procedures, setting reporting benchmarks.

In these applications several patterns emerged: (1) When combined with other methods, the KJ method can enhance effectiveness as an integrated tool; (2) Different stakeholder participation has improved the effectiveness of the framework; (3) Its collaboration and visibility enable the establishment of team consensus, which a single coding method cannot achieve. However, there are still gaps: there is a lack of standardized criteria for evaluating the KJ-derived framework, and the optimal number of iterations has not yet been determined. Future methodological research should develop reporting guidelines and quality evaluation tools suitable for the application of the KJ method.

### 4.5 Focus on patient preferences to guide the development of research tools

Patient preferences—defined as the subjective expressions of patients’ needs, values, and expectations—constitute essential input for developing patient-centered tools. However, these preferences are often fragmented and context-dependent, making them difficult to translate directly into tool specifications. The KJ method serves as a critical mediating mechanism in this translation process: through its structured procedures of card grouping, theme refinement, and hierarchical organization, it systematically transforms scattered patient-derived qualitative data into coherent conceptual frameworks that directly inform tool design and functional prioritization.

Several studies exemplify this logic. To assess the level of Media Health Literacy among Korean adults, researchers elicited preferences regarding media health information use from the target population, and constructed affinity maps to organize these data. Based on the affinity map results, the European Association for Viewers Interests model was selected as the main framework, and the MeHlit scale was formed through debugging, including access, critical evaluation and communication dimensions (36). Here, patient preferences directly determined the dimensional structure of the final tool. Cole et al. (37) developed an Electronic Health care Tool for patients with hematological malignancies. Qualitative data were collected through semi-structured interviews to capture diverse user preferences regarding the prototype’s functionality and usability. Affinity maps were established for thematic analysis to synthesize these preferences, providing an accurate multi-subject demand orientation for tool optimization and ensuring the final design reflected actual user needs rather than developer assumptions. Similarly, researchers (38, 39) analyzed data related to children with Spina Bifida, their parents, and urological healthcare providers through affinity mapping and online collaborative activities. Preferences regarding continence management goals were systematically collected and organized, and the visualization process of the incontinence goal selection tool prototype was developed accordingly, with key features directly traceable to preference themes identified through the KJ process.

The common logic across these cases is clear: patient preferences serve as the raw input, the KJ method provides the structured analytical process, and research tools emerge as the tangible output—each tool component corresponds to a preference theme identified during the KJ procedure. Traditional quantitative research methods focus on statistical analysis and hypothesis testing, while the KJ method complements these by emphasizing the collation and induction of qualitative data, enabling researchers to more deeply understand the subjective experiences that shape patient preferences and to develop tools that are genuinely responsive to patient needs.

## 5 Critical appraisal of current applications

Despite the increasing application of KJ method across nursing domains, several methodological concerns warrant attention. First, the majority of included studies employed small, purposive samples, which, while appropriate for qualitative exploration, raises questions about the transferability of their findings to broader nursing populations. Second, most studies did not report inter-rater reliability or consensus procedures during the card grouping and labeling stages, leaving the objectivity of the derived themes uncertain. Third, few studies adopted triangulation strategies—such as combining KJ-derived findings with quantitative validation or independent expert review—to strengthen the credibility of their conclusions. Fourth, while the KJ method is often described as a “team-based” tool, several studies did not clearly specify the composition of the grouping team or the decision-making process for resolving disagreements, which limits the replicability of their procedures. These limitations suggest that the current evidence base, while informative, remains preliminary and calls for more rigorous methodological reporting and validation in future applications. In addition, as a narrative review, our literature search was not exhaustive, and the selection of studies may have been influenced by the authors’ familiarity with specific research areas and databases.

## 6 Enlightenment and Prospect

This paper systematically combs the core operation process, application strategy and precautions of KJ method, comprehensively summarizes its application status in nursing education, nursing management, clinical nursing and nursing research, and deeply analyzes the application fitting and practical application effect of KJ method in various nursing scenarios, revealing its core value as a creative problem solving method in the nursing field. With the advantage of systematic qualitative information analysis, KJ method can effectively solve all kinds of complex and scattered qualitative problems in the nursing field and provide an important method support for the development of nursing discipline. However, there are still some problems in its application in the nursing field, such as lack of standardization, lack of system of application strategy, and insufficient integration with modern technology. Combined with the development trend of nursing discipline, the future development direction of KJ method in the nursing field is put forward as follows:

### 6.1 Deepen interdisciplinary integration and expand the application boundaries of methods

As a creative problem-solving method, KJ method has strong interdisciplinary application potential. In the future, it is necessary to further deepen its integration with nursing and other disciplines. On the one hand, it promotes the deep integration of KJ method and evidence-based nursing. In the development of clinical decision support system, KJ method integrates patient preferences, clinical practice experience and the best evidence to achieve personalized nursing and promote the transformation of evidence to clinical practice. On the other hand, the combination of KJ method with Artificial Intelligence (AI), big data and other modern technologies will become an important development trend. The use of natural language processing technology to analyze the qualitative data collected by KJ method can more efficiently explore the hidden laws in nursing phenomena. Big data technology was used to expand the information collection scope of KJ method and improve the comprehensibility of analysis results. With the advancement of technology and the deepening of interdisciplinary cooperation, KJ method will play a greater role in nursing education, management, clinical practice and research, and promote the development of nursing discipline to the direction of specialization, precision and internationalization.

However, several limitations and potential risks must be acknowledged when integrating the KJ method with emerging technologies. First, AI-based qualitative data analysis, while efficient, may introduce algorithmic bias (40) and misinterpret contextual nuances inherent in nursing narratives, as natural language processing models are often trained on general corpora rather than domain-specific clinical texts. Second, the use of big data raises significant concerns regarding patient privacy (41, 42) and data security, particularly when sensitive health information is processed through cloud-based platforms or third-party AI services. Third, there is a risk of over-reliance on automated analysis, which may diminish the essential humanistic and intuitive aspects of qualitative inquiry that are central to nursing practice—the very strengths that make the KJ method valuable. Fourth, the validity and reliability of AI-assisted KJ outcomes remain under-investigated, and the absence of standardized evaluation criteria for technology-mediated qualitative analysis poses challenges for evidence-based decision-making.

Therefore, while embracing technological innovation, future research should prioritize the development of nursing-specific AI training datasets, establish clear ethical guidelines and privacy protection protocols, and maintain a balanced approach that preserves the critical role of human judgment in the KJ process. With the advancement of technology and the deepening of interdisciplinary cooperation, the KJ method will play a greater role in nursing education, management, clinical practice, and research, and promote the development of the nursing discipline toward specialization, precision, and internationalization, provided that these challenges are adequately addressed.

### 6.2 Expand the application scenario to meet the development needs of nursing discipline

With the aggravation of the aging society, community nursing and home nursing have become important development directions of the nursing discipline, and the application of KJ method in this field has great potential (30, 43, 44). In view of the health management needs of home-based elderly care patients, KJ method can be used to integrate multiple perspectives of patients, family members and community medical staff, and build a comprehensive service model covering disease monitoring, psychological support and rehabilitation guidance (45, 46), so as to improve the service quality of community and home care. At the same time, interdisciplinary integration offers significant potential to advance the application of the KJ method in nursing. As Yan Ji et al. (47) noted, nursing cross-disciplinary research has formed three major directions: nursing with medicine and life sciences, nursing with information science and engineering, and nursing with social sciences and humanities. Specifically, psychology can contribute theoretical frameworks for understanding patient cognition, emotions, and behavioral responses, informing more nuanced theme interpretation when analyzing qualitative data related to patient experiences (48). Sociology provides perspectives on social support networks, family dynamics, and healthcare system structures, enabling the KJ method to better capture the social determinants of health that influence nursing outcomes (47). Education contributes pedagogical theories and curriculum development expertise, which can enhance the design of KJ method-based training programs for nursing students and in-service nurses (12, 13). Information science facilitates the digital transformation of the KJ method, enabling online card sorting platforms and automated qualitative data analysis tools that overcome the time and space limitations of traditional paper-based approaches.

The pathway for interdisciplinary integration can be operationalized at three levels: theoretical integration, where concepts from partner disciplines inform the analytical framework; methodological integration, where the KJ method is combined with other research techniques (e.g., psychological scales, sociological network analysis) to triangulate findings; and practical integration, where interdisciplinary teams co-facilitate KJ sessions, bringing diverse expertise to the card grouping and interpretation process. Such integration yields more comprehensive, context-sensitive solutions, such as community care models that simultaneously address patients’ emotional, social, and clinical needs (45, 46). However, challenges remain (49), including differences in disciplinary terminology and conceptual frameworks, varying epistemological assumptions, and logistical difficulties in coordinating cross-disciplinary teams (47). Future research should explore structured protocols for interdisciplinary KJ facilitation to ensure transparency and reproducibility.

### 6.3 Strengthen methodological innovation and improve the standardized application system

Based on the methodological gaps identified across the reviewed studies, four specific aspects of KJ method application require standardization.

First, team composition and consensus procedures. Studies vary in who participates in card grouping and how disagreements are resolved, yet most do not report consensus rules. Standardization should require: (a) reporting of team composition (number, backgrounds); (b) specification of consensus rules (unanimous agreement, majority vote, or iterative discussion); and (c) documentation of how disagreements were handled. Second, data unit preprocessing. Studies differ in how data are distilled into cards—using verbatim quotes, paraphrased summaries, or synthesized statements—affecting theme granularity and comparability. Standardization should recommend clear criteria for defining meaningful data units and ensuring consistency across researchers. Third, minimum reporting standards. Many studies lack essential details—grouping iterations, handling of “orphan” cards, respondent validation, or procedural modifications. Standardization should require reporting of: (a) grouping iterations and stopping criteria; (b) handling of ambiguous cards; (c) procedural modifications and rationales; and (d) respondent validation or expert review. Fourth, quality evaluation criteria. Unlike content analysis or thematic analysis, KJ method lacks established validity benchmarks. Standardization should guide the development of KJ-specific appraisal tools, including indicators such as thematic saturation, inter-coder agreement, and expert validation of hierarchical structures.

In addition, methodological innovation should focus on: developing digital KJ tools to overcome time and space constraints; integrating KJ method with quantitative methods for mixed-method validation (50, 51); and establishing a nursing-specific application guide incorporating the four aspects above to improve the scientific and normative application of KJ method in nursing practice.

### 6.4 Focus on the improvement of nurses’ core competence and promote the two-way transformation of nursing practice and research

With the increase of nursing complexity, nurses need to have higher information integration and problem solving skills. KJ method can be used as a tool for nurses’ critical thinking training (52). In the training of nursing case analysis and clinical problem solving, KJ method can guide nurses to systematically analyze patient information and clinical problems, formulate comprehensive nursing plans, and improve nurses’ structured thinking and problem solving ability.

This translation follows a clear six-stage cyclical pathway. First, nurses encounter complex clinical situations daily—unmet patient needs, communication difficulties, or workflow inefficiencies—which are often perceived as isolated impressions rather than clearly defined problems. Second, through KJ method’ s card-based grouping and diagramming, these scattered clinical observations are structured into coherent thematic frameworks, revealing patterns and root causes that might otherwise remain hidden. Third, these structured framew (53)orks are directly translated into research questions, quality improvement targets, or intervention prototypes, ensuring that priorities are grounded in authentic clinical realities. Fourth, these questions are investigated using appropriate qualitative, quantitative, or mixed methods to generate empirical evidence addressing the identified problems (44). Fifth, the generated evidence is synthesized and translated into actionable practice recommendations—a process in which KJ method can be reapplied to integrate research findings, clinical guidelines, patient preferences, and implementation feasibility data. Sixth, these recommendations are implemented and evaluated in clinical settings, generating new clinical experiences that initiate subsequent analytical cycles (11). This process is thus bidirectional and iterative: clinical practice informs research through problem identification and structuring, while research informs practice through evidence translation and implementation (54, 55).

In addition, KJ method was included in continuing nursing education courses, so that more nurses could master the application process and strategy of this method through systematic training, help in-service nurses improve their scientific research ability, especially qualitative research ability, and promote nurses to transform problems in clinical practice into research topics. At the same time, the research results were sorted out and applied to clinical practice through KJ method. To realize the two-way transformation of nursing practice and research, and improve the overall literacy of the nursing team and the quality of nursing service.

## 7 What is known?

- As a creative qualitative problem solving method, KJ method has been increasingly used in the field of nursing in recent years, covering many aspects such as nursing education, management, clinical practice and research.
- In nursing education, KJ method was used in the construction of teaching content framework and teaching model optimization; In nursing management, it helps to optimize nurse–patient communication and excavate nurses’ core competence. In clinical nursing, KJ method improves patients’ health management and nurses’ clinical ability. In nursing research, as a qualitative research tool, it promotes the development of theoretical framework and research tools.
- KJ method helps nursing staff to accurately understand problems and discover internal rules by systematically organizing and analyzing qualitative information, and its advantages have been widely recognized in the nursing field.

## 8 What is new?

- This paper systematically reviewed the comprehensive application status of KJ method in nursing education, management, clinical nursing and research for the first time, and analyzed its application fit with each nursing scenario, specific application strategies and effects in detail, so as to provide an important reference for the standardized application of KJ method.
- The existing problems in the application of KJ method in the nursing field, such as insufficient standardization, unformed application strategy system, and insufficient integration with modern technology, were analyzed in depth, which provided a clear improvement direction for future research.
- Combined with the development trend of nursing discipline, this paper puts forward four future development directions of KJ method in the nursing field: deepening interdisciplinary integration, expanding application scenarios, strengthening methodological innovation, and focusing on the improvement of nurses’ core competence, which provides new ideas for the sustainable development and innovative practice of KJ method.
